# Sleep Quality Is Associated with Vitamin B12 Status in Female Arab Students

**DOI:** 10.3390/ijerph18094548

**Published:** 2021-04-25

**Authors:** Sara Al-Musharaf, Alanoud Alabdulaaly, Hanadi Bin Mujalli, Hatoun Alshehri, Hind Alajaji, Rania Bogis, Ruyuf Alnafisah, Shaden Alfehaid, Hala Alhodaib, Alice M Murphy, Syed Danish Hussain, Shaun Sabico, Philip G McTernan, Nasser Al-Daghri

**Affiliations:** 1 Department of Community Health Sciences, College of Applied Medical Sciences, King Saud University, Riyadh 11451, Saudi Arabia; Alanoudibrahim1997@hotmail.com (A.A.); Hanadi.binmujalli@gmail.com (H.B.M.); Hatounalshehri@gmail.com (H.A.); Hindalajaji6@gmail.com (H.A.); Rbogis96@gmail.com (R.B.); Rynafisah@gmail.com (R.A.); Shadenalfehaid22@gmail.com (S.A.); halhodaib@ksu.edu.sa (H.A.); 2 Department of Biosciences, School of Science and Technology, Nottingham Trent University, Nottingham NG1 8NS, UK; alice.murphy@ntu.ac.uk (A.M.); philip.mcternan@ntu.ac.uk (P.M.); 3 Chair for Biomarkers of Chronic Diseases, Biochemistry Department, College of Science, King Saud University, Riyadh 11451, Saudi Arabia; shussain@ksu.edu.sa (S.D.H.); ssabico@ksu.edu.sa (S.S.); ndaghri@ksu.edu.sa (N.A.-D.)

**Keywords:** sleep difficulties, PSQI, poor sleep, sleep duration, serum vitamin B12, dietary vitamin B12

## Abstract

Studies have explored how vitamin B12 status affects sleep among elders and children, but this remains to be investigated among young adults. We used the Pittsburgh Sleep Quality Index (PSQI) to assess the association between serum vitamin B12 and sleep among female college students in Saudi Arabia. In this cross-sectional study, we enrolled 355 participants (age (years), 20.7 ± 1.5; body mass index, 23.6 kg/m^2^ ± 5.2) at King Saud University, Riyadh, Saudi Arabia. Fasting blood samples were analyzed regarding the serum vitamin B12 and blood lipids. Anthropometric, socio-demographic, clinical history, stress, physical activity, and dietary data were collected. We assessed the sleep statuses of the participants using the PSQI. Around 72% of the participants were “poor” sleepers (PSQI > 5). Subgroup analysis within the tertiles showed that participants with higher vitamin B12 in the second and third tertiles reported better scores for sleep quality (B ± SE = −12.7 ± 5.6, *p* = 0.03; B ± SE = −32.7 ± 16.4, *p* = 0.05, respectively) and also reported a lower use of sleep medication (B ± SE = −21.2 ± 9.9, *p* = 0.03, in the second tertile only), after adjusting for the waist–hip ratio and stress. However, sleep was not found to be directly associated with either serum vitamin B12 or dietary vitamin B12. In conclusion, the serum vitamin B12 results show that the participants with higher vitamin B12 in the second and third tertiles reported better scores on the sleep quality scale and a lower use of sleep medication. However, no such associations were observed with the overall PSQI. More studies with larger sample sizes are needed to establish a direct relationship between sleep and vitamin B12.

## 1. Introduction

Sleep is crucial for regulating metabolic, cognitive, and psychological functions necessary for maintaining and promoting health [1,2]. Sleep disturbances are increasing in both developing and developed societies, leading to a pressing public health problem among the general population [3,4]. Sleep difficulties may be more pronounced within certain populations, such as those who are pregnant and the elderly [5]. They are also more common in females than in males [6].

College students are vulnerable to sleep difficulties due to the academic and social demands in this age group [7]. Studies have highlighted a sharp increase in sleep difficulties from around 27% in 1982 to around 68% in the last decade among United States college students from multiple universities [8]. Studies in Saudi Arabia have shown a similar high prevalence of poor sleep quality, reaching around 70% [4,9,10], with a higher percentage among females [4]—a rate that is particularly alarming for college students, whose cognitive performance directly affects their academic success [1].

Long-term sleep difficulties are associated with a wide range of health burdens, including obesity [11], diabetes [12,13] and abnormal glycated hemoglobin [14], metabolic syndrome [15], and cardiovascular diseases [16]. Poor sleep quality has also been associated with inflammation [17,18], cancer [19], and an increased mortality risk [20]. Several risk factors for poor sleep have been proposed, such as age, ethnicity [21], and sociodemographic status [22]. Studies have also found associations between worse sleep and a longer dinner-to-bed time [21], alcohol consumption [23], and excess macronutrient intake [24].

Studies on the impact of micronutrient deficiencies on sleep have been limited [25,26,27]. Most studies on vitamin B12 have focused on its dietary intake, and few, on its serum levels [25,26,27]. Measuring the latter is important, as the absorption of the vitamin B complex is affected by many dietary and circulatory factors [28]. As such, measuring only the dietary intake of vitamin B12 can be misleading.

Vitamin B12, or cobalamin, is an essential vitamin that the body cannot manufacture and that must be supplied from dietary intake [29,30,31,32]. It is known for its essential role in the functioning of the nervous system [29,30,31,32]. The spectrum of consequences of B12 deficiency (defined as <148 pmol/L) ranges from mild fatigue and anemia to severe neurological impairment [29,30,31,32]. As such, it is a micronutrient of interest when considering associations with sleep difficulties.

However, studies investigating connections between serum vitamin B12 and sleep are scarce [33,34,35]. To our knowledge, only two studies have investigated the relationship between sleep difficulties and serum vitamin B12; one found an independent inverse relationship between serum vitamin B12 levels and sleep duration in adults [35], and the other found that low serum levels of vitamin B12 were associated with lower sleep efficiency in children [33]. However, the first studied the general population, including elderly patients [35], and the second studied children with disease [33].

Young females of child-bearing age are particularly vulnerable to sleep difficulties; however, few studies have been conducted on this cohort, especially in Middle Eastern countries. This is important, as sleep problems during pregnancy are associated with increased health problems [5]. We believe that ours is the first study to assess sleep difficulties among college students from a variety of university departments in Riyadh; similar local studies have focused exclusively on medical students [4,9,10].

In Saudi Arabia, the prevalence of serum vitamin B12 deficiency has been found to vary among populations with comorbidities and measured at 7.8% among individuals with type 2 diabetes mellitus [29] and 26% among patients with partial gastrectomy [30]. This study is also the first to address the relationship between sleep difficulties and serum vitamin B12 specifically in a young, healthy population. This work aimed to assess whether lower vitamin B12 serum levels are associated with poorer sleep quality in an apparently healthy female population while controlling for most confounding factors, including age, body mass index (BMI), physical activity, and stress.

## 2. Materials and Methods

### 2.1. Study Population

A total of 355 healthy individuals consented to and participated in this observational cross-sectional study. The participants were randomly selected from different departments at King Saud University (KSU). A sample size calculation was carried out before the study was conducted. Based on the differences in serum vitamin B12 levels according to sleep duration as reported in a previous study with an effect size of 0.217 [33], the total required sample size for a 95% confidence interval (CI) and 5% level of significance was determined to be 278. To account for a 25% nonresponse rate, we enrolled 355 participants.

### 2.2. Inclusion and Exclusion Criteria

To be included in the study, participants needed to be healthy, non-pregnant female Saudi college students at KSU, aged 19–30 years. The study excluded non-Saudis, pregnant women, and women diagnosed with any of the following: sleep or psychiatric disorders; gastrointestinal disorders; significant proteinuria or amyloidosis; arthritis; anemia; enlarged tonsils or adenoids; malabsorption; comorbid chronic diseases, such as thyroid disorders, diabetes mellitus, malignancies, or chronic obstructive pulmonary disease; or a history of metabolic disorders. We also excluded participants who had taken vitamin B12 supplements or medications with known effects on serum vitamin B12 levels.

### 2.3. Data Collection

The data were collected between January and March 2019. Students from a number of colleges within KSU met the criteria and consented to take part in the study. The participants gave permission for data collection and to have their blood stored in a biobank in the laboratories of the Chair for Biomarkers of Chronic Diseases (CBCD). The participants had the option to withdraw at any stage during the study. We obtained ethical approval from the institutional review board (IRB) of King Khalid University Hospital, Riyadh, before the start of the study (IRB number: E-19-3625).

#### 2.3.1. Anthropometric Assessment

Anthropometric data were collected according to standard procedures. Weight and height were recorded to the nearest 0.2 kg and 0.5 cm, respectively, using a Digital Pearson Scale (ADAM Equipment Inc., Oxford, MS, USA). To calculate the BMI (kg/m^2^), the weight (kg) was divided by the square of the height in meters (m^2^). Obesity was categorized according to the World Health Organization (WHO)’s cut-off of 30 kg/m^2^ [36]. The waist and hip circumference were measured using WHO procedures [37], and the waist/hip ratio (WHR) was obtained by dividing the mean waist circumference by the mean hip circumference. An InBody 770 (InBody, Cerritos, CA, USA) body composition analyzer was used on all the students to assess their percentages of body fat.

#### 2.3.2. Questionnaire Assessment

We first interviewed participants using a generalized questionnaire [38] that included questions on sociodemographic data (major, semester level, family income, and marital status), family medical history, student medical history, and medication treatment history. The students were then given the following questionnaires: the Pittsburgh Sleep Quality Index (PSQI), the Global Physical Activity Questionnaire (GPAQ), and the Saudi Food and Drug Administration’s Food Frequency Questionnaire (FFQ).

##### Sleep Index

The participants’ quality of sleep was assessed using the validated PSQI, which was translated into Arabic [39]. This assessment of sleep quality over a one-month period contains seven components: the subjective sleep quality, sleep latency, sleep duration, sleep efficiency, sleep disturbance, use of sleeping medication, and daytime dysfunction. The scores for each component ranges from 0 to 3, with higher values indicating worse sleep quality [40]. The additive total scores range from 0 to 21; scores above 5 indicate poor sleep quality, which increases in severity as the score rises [40]. We further classified the subjective sleep quality component scores as good (0 or 1) or poor (2 or 3). A sleep duration ≤7 h was considered short sleep [41].

##### Dietary Data Collection for Vitamin B12

A validated Saudi FFQ was used to measure vitamin B12 intake over the past year [42]. The questionnaire had been developed in the Arabic language. The FFQ lists 133 food items; asks questions regarding food frequency, the type of cooking fat, visible fat consumption, and the consumption of salt and vitamins [42]; and includes open-ended questions to gather information on food items not listed. The FFQ was analyzed using an Excel spreadsheet provided by Dr. Majed Alkhalaf (Microsoft Excel, Microsoft, Redmond, WA, 2003).

The food item values were based on the 1996 Saudi Food Composition Table, McCance and Widdowson’s Composition of Foods Integrated Dataset 2015, and the concise 2016 New Zealand Food Composition Tables, 12th edition [42,43,44]. A validated vitamin B12 questionnaire provided by Mearns and Rush was also used to assess the vitamin B12 intake [44]. The recommended dietary allowance (RDA) of vitamin B12 for adults (2.4 mcg/day [31]) was used as the threshold for adequate dietary intake.

##### Physical Activity Questionnaire

We used the GPAQ version 2.0, translated into Arabic [45], to assess physical activity; the same instrument was previously used on a college-aged Saudi population [46]. The questionnaire covers several components of physical activity, such as intensity, duration, and frequency. It also assesses three domains in which physical activity may be performed: occupational physical activity, transport-related physical activity, and physical activity during discretionary or leisure time [45].

##### Stress Questionnaire

The Perceived Stress Scale-10 (PSS-10) is a 10-item instrument developed by clinicians and is one of the instruments most commonly used to quantify stress in investigations of psychosomatic and somatic complaints [47]. We used the recent Arabic-language form of the scale, which asks about situations that took place during the previous month. Each item uses a five-point Likert-type scale, ranging from 0 (never) to 4 (very often); higher scores indicate more severe perceived stress [48].

#### 2.3.3. Biochemical Assessment

Blood samples were stored as serum and whole blood at −80 °C pending analysis at the CBCD laboratories at KSU. The levels of serum vitamin B12 were measured with an electrochemiluminescent immunoassay using a Roche Cobas e411 immunoassay analyzer (Roche Diagnostics, Munich, Germany). The intra- and inter-assay coefficients of variation (CVs) were 2.9% and 4.1%, respectively. For this study, the data on serum vitamin B12 levels were divided into tertiles: low (≤333.0 pmol/L), middle (333.1–482.2 pmol/L), and high (≥482.3 pmol/L). The data were categorized based on the serum vitamin B12 clinical cut-offs into three groups: low (<221 pmol/L), normal (221–701 pmol/L), and excess (>701 pmol/L) [49]. Vitamin B12 deficiency was defined according to the WHO cut-off (<148 pmol/L) [49].

The serum total cholesterol (TC), high-density lipoprotein cholesterol (HDL-C), triglyceride (TG), and glucose levels were measured by colorimetric methods using an automated chemistry analyzer (Konelab, ThermoFisher, Finland). The intra- and inter-assay CVs were as follows: TC, 0.7% and 1.5%; HDL-C, 0.6% and 1.2%; TGs, 0.9% and 1.8%; and glucose, 0.8% and 2.6%, respectively. The LDL cholesterol (LDL-C) was calculated using the Friedewald formula [50].

### 2.4. Data Analysis

The data were analyzed using the SPSS statistical software version 23.0. The normality of all the quantitative variables was tested before analysis. Descriptive statistics (the means, standard deviations, medians, quartiles, frequencies, and percentages) were used to quantify the quantitative and categorical study and outcome variables. Student’s t-test was used for independent samples, when appropriate, to compare the mean values of quantitative outcome variables across the categories of study variables.

Appropriate nonparametric tests were used if the distributions of the variables showed skewed patterns. Analysis of covariance (ANCOVA) was used to adjust for covariates. Linear regression between sleep and serum vitamin B12 was performed in each tertile group with sleep components as dependent and serum vitamin B12 as independent variable. Bonferoni correction was applied for multiple comparison. Furthermore, logistic regression analysis was carried out to assess the relationship between vitamin B12 and sleep status. The relationship between vitamin B12 and sleep status was also examined using the clinical cut-offs for vitamin B12 (Table S1).

All the statistical tests, including the linear regression, logistic regression, and ANCOVA, used stress and WHR as covariates. Beta coefficients, after adjusting for covariates, were obtained for serum vitamin B12 and seven components of the PSQI along with the total PSQI scores in each vitamin B12 tertile, where higher scores for the sleep components represented poor sleep. *p*-values of <0.05 and 95% CIs are used to report the statistical significance and precision of the estimates.

## 3. Results

The mean age for all 355 participants was 20.7 ± 1.5 years. Most of the participants were single (99.7%); approximately 80% had family incomes >10,000 SAR/month. The mean BMI for all the participants was 23.6 ± 5.2, with 12.4% of participants classified as obese. The median level of serum vitamin B12 was 398.9 (305.8–534.6) pmol/L, and the dietary vitamin B12 intake was 6.9 (4.4–10.8) mcg/day (Table 1). The prevalence of clinical vitamin B12 deficiency (<148 pmol/L) was 0.6% (2/355).

In this study, the global PSQI score ranged from 1 to 16, with a median of 7, mean of 7.5, and standard deviation of 2.9. Among the seven sleep components of the PSQI, sleep duration received the worst rating, with a mean score of 1.8 ± 1.1, followed by sleep day dysfunction (1.7 ± 0.9), sleep latency (1.4 ± 0.9), sleep disturbance (1.1 ± 0.5), sleep quality (1.0 ± 0.8), sleep efficiency (0.6 ± 1.0), and sleep medication (0.1 ± 0.4). Among our sample, 71.8% (255/355) were poor sleepers (PSQI > 5), 82.8% (294/355) reported sleeping for ≤7 h per day, and 24% (83/355) had poor sleep quality.

There were no sociodemographic differences between the groups of poor and good sleepers, or any difference in BMI. We observed the WHR to be higher in poor sleepers than in good sleepers; the difference reached significance after adjusting for age, BMI, and physical activity (0.72 ± 0.06 vs. 0.70 ± 0.06, *p* = 0.048).

The median dietary intake of vitamin B12 was slightly higher in good sleepers than in poor sleepers, but this difference was found to be non-significant after adjustment for confounding factors (7.4 vs. 6.8 mcg/day; *p* = 0.21) (Table 1). The median coffee intake was found to be significantly higher in the poor sleepers; the difference remained significant after adjustment for confounding factors (120.0 (38.7–270.0) vs. 90.0 (25.8–150.0) mL/day; *p* = 0.049).

A slight difference was found between the two groups in terms of level of physical activity, with the good sleepers having a higher GPAQ score (Table 1). The mean stress score was significantly higher in poor sleepers compared with good sleepers, with a significant difference retained after adjustment for confounding factors (20.3 ± 5.8 vs. 16.8 ± 6.4; *p* = <0.001) (Table 1).

The levels of serum vitamin B12 were slightly lower in the poor sleep group than in the good sleep group; however, this difference was insignificant (396.6 [305.2–533.1] vs. 436.2 [313.0–535.6] pmol/L, *p* > 0.05) (Table 1). The PSQI scores were also analyzed using the vitamin B12 tertiles and clinical cut-offs; however, no differences in the means were observed (Supplementary Materials Tables S1 and S2).

Logistic regression analysis for the serum vitamin B12 tertiles and poor sleep status also confirmed that there was no association between sleep and vitamin B12 (Table 2). The relationship between serum vitamin B12 status and sleep status was also examined using the clinical cut-offs for vitamin B12 (Supplementary Table S3). The results suggest that low (<221 pmol/L) and excess (>701 pmol/L) vitamin B12 were not associated with sleep status as compared to normal vitamin B12 levels (221–701 pmol/L) (Supplementary Materials Table S3).

Subgroup analysis within the vitamin B12 serum tertiles showed that serum vitamin B12 was associated with some of the components of the PSQI. Linear regression between sleep and serum vitamin B12 was performed in each vitamin B12 tertile group after controlling for covariates (Table 3). Serum vitamin B12 was positively associated with a higher (worse) sleep duration score and total PSQI score (*p* = 0.03 and *p* = 0.05, respectively) in the first tertile. This suggests that participants with high serum vitamin B12 in first tertile experienced short sleep durations and overall poor sleep.

The subjective sleep quality component was negatively associated with the serum vitamin B12 level in both the second tertile (333.1–482.2 pmol/L) (*p* = 0.03) and third tertile (≥482.3 pmol/L) (*p* = 0.05) after adjusting for WHR and stress (Table 3). This suggests that subjects with higher serum vitamin B12 in the second and third tertiles reported better scores for the sleep quality components. Participants with higher serum vitamin B12 levels in the second tertile also reported a lower use of sleep medication (*p* = 0.03) (Table 3).

Linear regression between the sleep components and the serum vitamin B12 clinical cut-offs, after controlling for covariates (see Supplementary Materials Table S4), did not show any significance with the sleep components except for sleep latency. The sleep latency component was negatively associated with the serum vitamin B12 (B ± SE = −45 ± 4, *p* = 0.013, in the excess vitamin B12 group only). This suggests that participants with excess serum vitamin B12 took less time to fall asleep.

## 4. Discussion

To our knowledge, this is the first study to assess the association between a sleep index and serum vitamin B12 among apparently healthy, young female adults. This study showed that there was no direct relationship between serum vitamin B12 and sleep among the whole sample. However, subgroup analysis showed that serum vitamin B12 was independently positively correlated with sleep quality and the sleep medication components of the PSQI in the second and third tertiles. In addition, participants with excess serum vitamin B12 (>701 pmol/l) took less time to fall asleep. However, serum vitamin B12 also showed a negative association with the sleep duration and overall sleep status in the first tertile.

Among 355 apparently healthy Arab female college participants, we noted high PSQI scores (PSQI scores >5, indicating poor sleep) for 255 (71.8%) students. Two hundred ninety-four students (82.8%) reported sleeping ≤7 h per day. Studies of medical students in Saudi Arabia have reported similar results, with high PSQI scores being found among 63% to 76% of students [9,10] and students reporting an average of 5.65 to 6 h of sleep per day [9,10]. These Saudi figures are higher in terms of the total PSQI and lower in terms of the sleep duration than those for other countries in the Middle East and elsewhere [8,51,52]. The reported variations between countries may be influenced by differences in socioeconomic status and cultural habits.

Prior studies correlating serum vitamin B12 with sleep indices are rare but have reported significance among different sleep components [33,35]. Beydoun and colleagues conducted a cross-sectional study using the National Health and Nutrition Examination Survey with 2459 adults aged 20–85 years, adjusting for confounders, including the BMI, and found an independent inverse relationship between the serum vitamin B12 levels and sleep duration in adults [35]. A cross-sectional study of 63 children with familial Mediterranean fever measured their serum vitamin B12 in relation to sleep using the PSQI and found that children with low serum levels of vitamin B12 had lower sleep efficiency [33].

No correlation was found with other sleep indices, including the total PSQI and sleep duration. One study [33] examined a small, nonhealthy cohort, while another included a wide age range that included elderly participants [35]. Possibly related is a controlled clinical trial by Takahashi et al. studying vitamin B12 supplementation in patients with delayed sleep phase syndrome, which reported a significant short-term improvement of the sleep–wake cycle in supplemented patients [53]. Maeda and colleagues also suggested that supplementation with vitamin B12 (methylcobalamin) was of therapeutic benefit in treating sleep–wake disorders, as it helps to regulate circadian rhythms [54].

Our study found that participants with higher serum B12 concentrations, in the second and third tertiles, reported better sleep quality than those with lower serum B12 concentrations. Participants with higher serum vitamin B12 levels in the second tertile also reported a lower use of sleep medication. These results are comparable to those of previous studies [33,53,54].

On the other hand, our study observed that participants with high serum vitamin B12 in the lowest tertile reported shorter durations of sleep and also scored poorly on the PSQI. These results are inconsistent with the previous literature [33,35]. Researchers have proposed that a low serum level of vitamin B12 disrupts methylation in the central nervous system and could lead to neurological and psychiatric disorders [55]. Serum vitamin B12 was shown to modulate human melatonin secretion through the remethylation of homocysteine to methionine, which cooperates with hydroxyindole-O-methyltransferase in the synthesis of melatonin [56].

Alternatively, Mayer et al. suggested that vitamin B12 caused changes in the visual analogue scale scores, providing evidence of its alerting effects, with significant findings of improved sleep as well as improved concentration and freshness throughout the day [57]. Finally, low serum vitamin B12 levels may affect the body’s ability to manufacture sufficient numbers of red blood cells to effectively transport oxygen throughout the body, leading to a feeling of tiredness and weakness [29,30] and, possibly, eventually, to decreased overall sleep quality.

The opposing nature of the associations found in our study between the lowest and other tertiles may be explained by the low number of participants with vitamin B12 deficiency among our sample. Another explanation may be other micronutrient deficiencies or biochemical parameters that may affect the sleep duration among the group with the lowest vitamin B12 levels.

A meta-analysis showed that the micronutrient status was linked to sleep duration, with sleep duration positively associated with the Fe, Zn, and Mg levels and inversely related to the serum Cu and K levels [34]. The type of population, students with a high level of stress, and high caffeine consumption may play roles in the correlation between vitamin B12 deficiency and sleep disorders, as they implicate many other confounding factors. However, our study attempted to assess the majority of confounders to limit this bias. Finally, the young ages of the participants with healthy glucose levels, normal lipid profiles, and normal BMIs may play a role in the significance of the whole sample.

We found that the vitamin B12 intake of good sleepers (PSQI score ≤5) was slightly higher than that of poor sleepers, but this difference was not statistically significant. Two Japanese studies, one among 112 women aged 19–36 years old and the other among 1902 healthy adults aged 30–60 years, reported that a lower intake of vitamin B12 was likely to correlate with a later sleep period [27] and with a shorter sleep duration [26]. By contrast, Jahrami et al. conducted a case–control study among 96 Bahraini controls and 96 patients with depression and found that the total PSQI score was positively associated with the vitamin B12 intake in healthy adults [58].

Altered absorption with increased age may partly explain the differing results from a cohort with a wider age range [25,57]. Age-related differences in the absorption of protein-bound vitamin B12 included a higher prevalence of atrophic gastritis, a decrease in acid-pepsin secretion by the gastric mucosa, and bacterial overgrowth in the gastrointestinal tract [59]. In our young and healthy population, we found that a higher dietary intake of vitamin B12 corresponded to better sleep.

We observed that poor sleepers had worse stress scores and higher WHRs than good sleepers after adjustment for confounders. The bidirectional interaction between stress and poor sleep, along with sleep disorders, has been well established in the literature [60,61]. Almojali et al. demonstrated a statistically significant association between poor sleep quality and stress among medical students attending King Saud bin Abdulaziz University for Health Sciences in Riyadh, Saudi Arabia [9].

The association between obesity and poor sleep is also well established in the literature [62,63]. Rahe et al. (2015) highlighted that poor sleep quality was associated with obesity and high body fat [63], and a cross-sectional study indicated that centrally obese women had lower sleep efficiency and less slow wave sleep than their less obese counterparts [62]. The association between obesity and poor sleep we observed in our study did not reach statistical significance; however, we did observe significance in the relationship with the WHR.

The limitations of the methodology and study outcomes include the cross-sectional design, which precluded causal inference through the directionality of relationships. Second, although serum vitamin B12 measurement provides a suitable assessment of the general vitamin B12 status in population surveys, plasma methylmalonic acid, homocysteine, or holotranscobalamin II can also be used. Future studies could seek to confirm our findings using these additional biochemical measurements.

Third, there was a chance of recall bias during the data collection, as the PSQI for sleep assessment and the other questionnaires used (FFQ and GPAQ) depended on subjective reporting. Fourth, the young ages and lack of metabolic diseases of the participants may have contributed to the insignificant results; due to a limited budget, we were not able to follow up with the participants. Thus, future studies should involve older participants. Studies using objective tools for sleep assessment and older age groups will be needed to further evaluate the relationship between sleep and vitamin B12.

Despite the limitations, our study is the first to assess the association between serum vitamin B12 and sleep indices of the PSQI in apparently healthy young female adults in Saudi Arabia. We accounted for more confounding factors than prior studies to rule out influences on the vitamin B12 levels, such as those from the dietary intake, BMI, age, stress, and physical activity.

## 5. Conclusions

In summary, we observed that there was no direct relationship between vitamin B12 and sleep. However, higher vitamin B12 levels were associated with better sleep quality and lower use of sleep medication according to the subgroup analysis. Further studies are needed to establish the relationship between vitamin B12 and sleep status.

## Figures and Tables

**Table 1:** General characteristics according to sleep status.

	Overall	Good Sleep	Poor Sleep	*p*-Value	*p*-Value **
N (%)	355	100 (28.2)	255 (71.8)		
Age (years)	20.7 ± 1.5	20.8 ± 1.5	20.7 ± 1.6	0.74	
Family income (>10,000 SAR/month)	281 (79.8)	77 (77.0)	204 (80.0)	0.53	
Single	345 (99.7)	97 (97.0)	248 (97.3)	0.79	
Working part time	11 (3.2)	2 (2.0)	9 (3.5)	0.74	
Educational level, semester					
1–5	166 (46.8)	43 (43.0)	123 (48.2)	0.28	
6–9	176 (49.6)	51 (51.0)	125 (49.0)		
≥10	13 (3.7)	6 (6.0)	7 (2.7)		
Anthropometrics					
Body mass index (BMI) (kg/m^2^)	23.6 ± 5.2	23.2 ± 4.7	23.8 ± 5.4	0.38	0.28 ***
Waist–hip ratio	0.70 ± 0.1	0.70 ± 0.06	0.72 ± 0.06	0.06	
Fat (%)	36.9 ± 8.2	36.3 ± 8.4	37.1 ± 8.1	0.38	0.44
Obese *n* (%)	44 (12.4)	11 (11.0)	33 (13.0)	0.61	
Biochemistry					
Vitamin B12 (pmol/L) #	398.9 (306–535)	436.2 (313–536)	396.6 (305–533)	0.67	0.86
Vitamin B12 deficiency (<221 pmol/L)	21 (5.9)	6 (6.0)	15 (5.9)	0.97	0.91
Vitamin B12 deficiency (<148 pmol/L)	2 (0.5)	1 (1.0)	1 (0.4)	0.47	0.34
Glucose (mmol/L)	4.6 ± 1.0	4.7 ± 1.0	4.6 ± 1.0	0.37	0.45
Triglycerides (mmol/L)	0.8 ± 0.4	0.8 ± 0.4	0.8 ± 0.4	0.81	0.31
Total cholesterol (mmol/L)	3.8 ± 1.4	3.8 ± 1.4	3.8 ± 1.5	0.99	0.80
LDL cholesterol (mmol/L)	2.3 ± 1.1	2.4 ± 1.2	2.3 ± 1.1	0.92	0.56
HDL cholesterol (mmol/L)	1.0 ± 0.4	1.1 ± 0.4	1.0 ± 0.4	0.54	0.57
Dietary intake					
Vitamin B12 (mcg/day) #	6.9 (4.4–10.8)	7.4 (4.4–12.6)	6.8 (4.4–10.5)	0.17	0.21
Coffee (mL/day) #	104.1 (39–210)	90.0 (26–150)	120.0 (39–270)	0.009	0.05
Tea (mL/day) #	103.2 (33.6–240.0)	103.2 (33.6–240.0)	180.0 (67.2–240.0)	0.04	0.37
Physical activity					
Sitting (minutes/day) #	420 (240–600)	420 (270–600)	360 (240–600)	0.83	0.32
GPAQ score #	504 (160–1240)	522 (230–1240)	500 (160–1240)	0.77	0.90
PSS-10 score	19.3 ± 6.2	16.8 ± 6.4	20.3 ± 5.8	<0.001	

Data presented as the mean ± standard deviation (SD); # indicates non-normal variables; *p*-values were obtained from independent sample *t*-tests and the Mann–Whiney U test for normal and non-normal variables, respectively. ** indicates the waist/hip ratio (WHR) and stress were adjusted; *p* < 0.05 was considered significant. *** indicates *p*-values adjusted for stress. Saudi Arabia Riyals (SAR), Perceived Stress Scale (PSS-10), and Global Physical Activity Questionnaire (GPAQ).

**Table 2:** Association between vitamin B12 tertiles and poor sleep (Pittsburgh Sleep Quality Index (PSQI) > 5) status.

	Unadjusted		Adjusted	
	OR (95%CI)	*p*-Value	OR (95% CI)	*p*-Value
Tertile 1 ≤ 333.0 pmol/L	Reference			
Tertile 2 333.1–482.2 pmol/L	0.8 (0.5–1.5)	0.525	1.0 (0.5–1.8)	0.873
Tertile 3 ≥ 482.3 pmol/L	0.9 (0.5–1.6)	0.764	0.9 (0.5–1.7)	0.833

Odd ratios (ORs) and 95% confidence intervals (CIs) for the ORs were obtained using multivariate logistic regression analysis, taking poor sleep status as a dependent variable against serum vitamin B12 tertiles as the independent risk. *p* < 0.05 was considered significant. The adjusted model was adjusted for the WHR and stress.

**Table 3:** Correlation between sleep and serum vitamin B12 by tertile.

Tertile		Vitamin B12	Vitamin B12	Vitamin B12
Vitamin B12 (pmol/L)		≤333.0 pmol/L	333.1–482.2 pmol/L	≥482.3 pmol/L
Sleep Quality	Beta ± SE	4.6 ± 5.8	−12.7 ± 5.6	−32.7 ± 16.4
	*p*-value	0.43	0.03	0.04
Sleep Latency	Beta ± SE	6.6 ± 4.8	−2.0 ± 5.0	5.1 ± 14.7
	*p*-value	0.17	0.69	0.73
Sleep Duration	Beta ± SE	9.1 ± 4.2	-2.5	−14.1
	*p*-value	0.03	0.55	0.25
Habitual Sleep Efficiency	Beta ± SE	3.2 ± 4.8	−2.3 ± 4.6	14.1 ± 13.6
	*p*-value	0.51	0.61	0.30
Sleep Disturbance	Beta ± SE	−4.3 ± 11.3	−6.2 ± 9.4	−10.4 ± 27.6
	*p*-value	0.70	0.51	0.71
Sleep Medication	Beta ± SE	11.4 ± 14.3	−21.2 ± 9.9	30.3 ± 44.2
	*p*-value	0.43	0.03	0.49
Sleep Day Dysfunction	Beta ± SE	−1.5 ± 5.4	4.0 ± 5.0	−0.6 ± 13.8
	*p*-value	0.78	0.43	0.96
Total PSQI	Beta ± SE	3.0 ± 1.6	−2.4 ± 1.6	−3.2 ± 4.8
	*p*-value	0.05	0.17	0.50

Beta ± SE and 95% CIs were obtained using linear regression analysis, taking the sleep components as dependent variables against the serum vitamin B12 as the independent variable in each tertile. *p*-values were adjusted for the WHR and stress. Pittsburgh Sleep Quality Index (PSQI).

## Data Availability

The data from this study are available from the corresponding author on reasonable request.

## Supplementary Materials

The following are available online at https://www.mdpi.com/article/10.3390/ijerph18094548/s1: Table S1: Analysis of covariance between PSQI scores and serum vitamin B12 status, Table S2: Analysis of covariance between PSQI scores and serum vitamin B12 tertiles, Table S3: Association between serum vitamin B12 status and sleep status, Table S4: Correlation between sleep and serum vitamin B12 in terms of deficiency, normal and excess.

## Abbreviations

BMI: Body mass index; GPAQ: Global Physical Activity Questionnaire; IRB: Institutional review board; KSU: King Saud University; ORs: Odds ratios; PSQI: Pittsburgh Sleep Quality Index; RDA: Recommended dietary allowance; SFDA FFQ: Saudi Food and Drug Administration’s food frequency questionnaire; WHR: Waist-to-hip ratio; WHO: World Health Organization; PSS-10: Perceived Stress Scale-10; TC: Total cholesterol; HDL-C: High-density lipoprotein cholesterol; TGs: Triglycerides; CBCD: Chair for Biomarkers of Chronic Diseases.
