# Supplementary material for: Sleep Quality Is Associated with Vitamin B12 Status in Female Arab Students
Source: Int J Environ Res Public Health. 2021 Apr 25;18(9):4548. doi: 10.3390/ijerph18094548 (PMC8123316; doi:10.3390/ijerph18094548)
Supplement: Supplementary file 1 [file ijerph-18-04548-s001.zip › ijerph-1172355-SI.pdf]

Table S1. Analysis of covariance between PSQI scores and serum vitamin B12 status.

Vitamin B12 Status	Mean ± SE	*p*-value *
Low (<221 pmol/l)	7.1 ± 0.6	
Normal (221–701 pmol/l)	7.6 ± 0.2	0.63
Excess (>701 pmol/l)	7.4 ± 0.5	

Data presented as Mean ± SE; * indicates p-values adjusted for WHR and stress. Pittsburgh Sleep Quality Index (PSQI).

Table S2. Analysis of covariance between PSQI scores and serum vitamin B12 tertiles.

Vitamin B12 Status	Mean ± SE	*p*-value *
Tertile 1 (<333.05 pmol/l)	7.5 ± 0.3	
Tertile 2 (333.1–482 pmol/l)	7.7 ± 0.3	0.88
Tertile 3 (>482 pmol/l)	7.6 ± 0.3	

Data presented as Mean ± SE; * indicates p-values adjusted for WHR and stress. Pittsburgh Sleep Quality Index (PSQI).

Table S3. Association between serum vitamin B12 status and sleep status.

	Unadjusted		Adjusted	
	OR (95% CI)	P-value	OR (95% CI)	*p*-value
Normal (221–701 pmol/l)			Reference	
Low (<221 pmol/l)	0.9 (0.4–2.5)	0.880	0.9 (0.3–2.6)	0.897
Excess (>701 pmol/l)	0.9 (0.4–2.2)	0.863	1.0 (0.4–2.5)	0.977

ORs and 95% CIs for ORs were obtained using multivariate logistic regression analysis, taking poor sleep status as a dependent variable against serum vitamin B12 clinical cutoff groups as independent risk. P< 0.05 considered significant. The adjusted model adjusted for WHR and stress.

Table S4. Correlation between sleep and serum vitamin B12 by deficiency, normal and excess.

Vitamin B12 (pmol/L)		Low (N = 21) ≤221	Normal (N = 292) 221–701	Excess (N = 28) ≥702
Sleep Quality	B ± SE	−1.2 ± 11.4	7.8 ± 9.0	17.2 ± 31.6
	P-value	0.91	0.39	0.59
Sleep Latency	B ± SE	1.9 ± 10.8	−8.1 ± 7.8	−45.4 ± 17.0
	P-value	0.86	0.30	0.013
Sleep Duration	B ± SE	9.5 ± 7.4	1.8 ± 6.8	3.2 ± 16.1
	P-value	0.20	0.80	0.85
Habitual Sleep Efficiency	B ± SE	−0.3 ± 9.5	−3.1 ± 7.3	22.4 ± 20.6
	P-value	0.98	0.67	0.29
Sleep Disturbance	B ± SE	27.1 ± 26.7	8.4 ± 15.1	−88.1 ± 55.5
	P-value	0.33	0.58	0.12
Sleep Medication	B ± SE	37.3 ± 35.6	−15.1 ± 19.4	48.5 ± 49.5
	P-value	0.31	0.44	0.34
Sleep Day Dysfunction	B ± SE	11.1 ± 11.1	0.4 ± 7.8	2.0 ± 22.6
	P-value	0.33	0.96	0.93
Total PSQI	B ± SE	4.3 ± 3.5	−0.3 ± 2.6	−5.8 ± 8.5
	P-value	0.23	0.90	0.50

Beta ± SE and 95% CIs were obtained using linear regression analysis, taking sleep components as dependent variable against serum vitamin B12 cut off groups as independent risk. P-values are adjusted for WHR and stress. Pittsburgh Sleep Quality Index (PSQI).
